# Consideration of Some of the Functional Signs of Diseases of the Stomach1Read before the Roswell Park Medical Club, at the club rooms in the Mooney-Brisbane Building, October 7, 1901.

**Published:** 1901-12

**Authors:** J. H. Potter

**Affiliations:** Buffalo, N. Y.


### Consideration of Some of the Functional Signs of Diseases of the Stomach.[1]

By J. H. POTTER, M. D., Buffalo, N. Y.

IT IS not my intention to present anything new in considering the functional signs of diseases of the stomach, but to review our present knowledge of these signs. A consideration of the clinical history and physical signs are often attended with doubt as to the actual conditions present, but a consideration of the functional signs gives a more positive and direct knowledge of the work the stomach is capable of performing. A diagnosis made without a consideration of the functional signs is incomplete and lacks scientific accuracy.

1. Read before the Roswell Park Medical Club, at the club rooms in the Mooney-Brisbane Building, October 7, 1901,

The stomach acts as a reservoir, the action of which is to a certain extent self-regulating. It also has three functions to perform when food is introduced, secretion, churning and evacuating movement, and absorption. While it may be possible for an individual to live without a stomach, a variation from the normal condition in either of the above functions leads to more or less local and constitutional disturbance. It is well to consider the subject under four divisions. Secretion, motor function, absorption, and digestive work. The stomach, as you well know, secretes mucus, an acid, and two ferments. I will not take up your time by going into the anatomy of the stomach or considering the different cells engaged in secreting the different substances; it may be well, however, to know that the fundus secretes more acid and the pyloric portion more pepsin. The method of studying secretion at the present time is by giving what are called test-meals. These may be given and withdrawn at definite intervals, and by chemical examination, determining the various digestive substances present.

Various methods have been used at different times to obtain the stomach contents. Among these might be mentioned vomiting, the use of a silver cup which is attached to a thread, swallowed and allowed to remain for a short time and then withdrawn, also swallowing small pieces of sponge which are then withdrawn. None of these methods are as free from error as the stomach-tube, and the latter is about the only one generally employed.

The tube should be used in every case where a positive diagnosis cannot be made without it, and when no contra-indications exist. Some of the objectionable indications to the use of the tube, it may be well to consider in this connection. Among these are acute diseases of the throat and stomach, general peritonitis, perigastritis, advanced soft carcinoma and ulcer, also similar condition of the esophagus and existence of aneurism of the aorta. It is best to defer the operation in all cases of recent hemorrhage, old age, severe valvular disease or degeneration of the heart muscle, advanced renal disease, cyanosis; and when obstructed respiration is present, it is advisable to avoid the use of the tube. It also should rarely be introduced during pregnancy, and, lastly, the refusal of the patient to admit the use of the tube.

The best method of obtaining the stomach contents I have found to be the use of Turk's stomach evacuating aspirator. By this method only sufficient should be withdrawn for analysis. If this is not at hand, then the ordinary stomach-tube can be used, and by instructing the patient to strain and make an effort to vomit, one may be able to obtain some of the stomach contents. If it is desired to empty the stomach, the patient may be placed in the knee-chest position. If no contents are obtained by these methods, then a small quantity of water may be introduced, withdrawn and examined.

There are various forms of test-meals, but we shall consider those only which are most frequently used. Examination should be made in the condition which prevails when the stomach is doing its normal work. The simplest test-meal is that of Ewald and Boas. The patient is given in the morning on an empty stomach one roll and 350 c.c. of water. The bread should be taken in the mouth dry, thoroughly masticated and washed down with water. One hour after the beginning of the breakfast the contents should be removed. In normal condition, at the end of an hour we obtain from 30 to 50 c.c. of a yellowish homogeneous mixture, which is filtered with ease. The total acidity should be about 55, acid albumin acidity, 45, and the free HCl acidity about 10. Free HCl appears in thirty minutes, reaches its height in about an hour and then diminished to the end of gastric digestion. The stomach is empty in from two to two-and-one-half hours from the beginning of the breakfast.

A more elaborate test-meal is that of Germain Seé. This test-meal consists of 60 to 80 gm. of chopped beef free from fat and fibrous tissues, 100 to 150 gm. white bread and a glass of water. The contents are removed two hours after the beginning of the meal, which should not be eaten rapidly. The test-dinner of Riegel consists of plate of beef soup, 150 to 200 gm. beef steak and 50 gm. potatoes and a small roll. Food should be thoroughly masticated, and tough fibrous bits of meat removed and contents obtained three or four hours from the beginning of the meal.

The test-breakfast, test-dinner and test-meal reveal accurately and fully the digestive work of the stomach. The test-meal of Ewald and Boas does not always fully test the digestive power of the stomach, and it is frequently advisable to use one of the other test-meals. It is sometimes a good plan to use two of the different meals in the same case.

In consideration of the HCl it is at present the universally accepted theory that the hydrocloric acid is formed and given out in a free state by the border cells. The acid unites with the mucus, exfoliated cells, saliva found in the stomach, and inorganic carbonates of the food and saliva, and this part for the digestion of the food is lost. Another part unites with the proteids of the food and converts them into acid compounds, which with the aid of pepsin are built up into albumoses and peptones. After the affinities of the proteids are satisfied, then the excess of HCl remains free. This excess is limited by the normal stomach and when this limit is exceeded, or, not reached within the proper time for the particular test-meal, the acid secretion is abnormal. If we know the amount of free acid that should be present at a given time after a test-meal in a normal stomach, it will then be easy to determine whether we have a case of a diminishing amount of acid secretion.

Some of the methods for determining the amount of acid present are elaborate and fit only for laboratory use; but for the general practitioner it is necessary to have a method which is simple, short and fairly accurate. In this connection it would be well to consider the substances usually tested for, and the manner of determining whether they are present or not. Given a test-meal, the first thing to consider is its reaction. This is determined by the use of litmus paper. If acid in reaction, the kind of acid present has to be determined. If neutral or alkaline, then the test for free acid is not necessary.

Lactic acid will probably give the same reaction and if you wish perfect accuracy take 5 to 10 c.c. of filtrate with a double quantity of ether and shake in a test-tube, allowing it to stand for a few minutes until ether has separated; pour off etheral solution and put in hot $H_2O$ till ether has evaporated and test the remaining sediment.

The first test is for HCl. If HCl is not present, then either of the following acids, lactic, fatty or butyric. The simplest and easiest test for HCl is, first, to a few drops of stomach contents add one drop of phloro-glucin-vanillum; stir with glass rod, evaporate to dryness. If HCl be present a bright cherry color appears. To determine whether the lactic acid is present, to a 2 per cent. solution of carbolic acid add one drop of tincture chloride of iron. Add filtrate to this blue solution. If yellow color appears then lactic acid is present; if gray, fatty acid, while organic acids decolorise the carbolic solution. To determine the total acidity present add one drop pheol-ptyalin to 10 c.c. of filtrate, add drop by drop of this solution and read biuret for amount used. If biuret reads six, the total acidity equals sixty. To determine the amount of HCl. present multiply biuret reading by ten and that by .00365.

To determine the work of the ferments it is well to examine for pro-peptones and peptones. Ferment is determined by the following method. To the filtrate add equal amount of sat. solution NaCl. The degree of opalescence shows proportion of quantity of pro-peptone. If no precipitate is present add a drop or two of acetic acid; will get a turbid sediment if pro-peptone is present. To determine the peptones, filter the above solution, make slightly alkaline and add few drops of a 1 per cent. solution of sulphate of copper. If resulting purple, it shows peptones.

It may also be well to consider the two ferments. By some clinicians this method is not utilised, but it is of aid in determining the pathological condition present. Of the two, labferment and labzymogen are the first ones to consider. As you know, labzymogen is a specific which is secreted by the cells and by the action of hydrochloric acid present, labferment is formed. The labferment coagulates milk by the disintegration, of the casein. The coagulation differs widely from that of acids and takes place in the presence, but independently, of any free acid in the stomach. This takes place when the medium is neutral or weakly acid.

A simple test for labferment is to place three or four drops of the unfiltered and unneutralised gastric contents in a covered glass and to this is added 5 c.c. of sweet milk. If this is kept at a body heat in from ten to fifteen minutes coagulation takes place. The qualitative test for the labzymogen requires a special preparation of the gastric contents. Five c.c. of the contents are made slightly alkaline with 1 per cent. solution of sodium carbonate, to this is added about 2 c.c. of cynid calcium chlorid, and mixed with a quantity of sweet milk and kept in a body temperature; the alkalinisation has destroyed the ferment and the calcium chlorid will convert the labzymogen, if present, into labferment and the coagulation will take place in the usual time.

Now there may be three conditions present, first, labzymogen may be normal, variable or persistently diminishing. If this is nearly always present after the test-meal, while not absolute proof that no pathological condition is present, it is very much in favor of its absence. If the acid secretion and motor function are normal, then we can almost be positive that no pathological condition is present. If there is continuous excessive secretion of HCl., then the labferment is frequently present in greater quantity than the labzymogen. The rule which claims that normal milk curdling power of the gastric contents excludes all but the dynamic affections does not hold true in some cases of glandular gastritis, ulcer and complicated myasthenia.

A variable labsecretion is a common sign of incipient and mixed forms of gastritis or of myasthenia and obstructive stagnation or retention. The quantity varies because the interstitial inflammation varies and because the evacuation of the stomach contents is irregular. If the labsecretion is persistently diminished, then there is glandular disease and the degree of deficiency indicates the degree and diffusion of the gastritis, or it indicates glandular degeneration.

The second ferment is pepsin and pepsinogen. The pepsinogen is a substance which is secreted by the cells and pepsin is formed by the action of the free HCl. The quantity of pepsinogen is conditioned by the percentage of free hydrochloric acid, about 2.5 parts, a 1,000 being the most favorable strength. The organic acids also possess this power of conversion.

The simplest form or method of testing this condition is that of Leube. Two tests are made, the one with the gastric contents alone and the other with the addition of pepsin. If the latter dissolves an equal part of albumin more rapidly than the former, the pepsin is deficient. Boas compares the rapidity of the solution of albumin by the gastric contents to be tested with the rapidity of the solution by the same quantity of albumin by the normal contents. Pepsin may be formed in excessive, normal or subnormal quantities. A continuous normal pepsinogen is a good sign of the integrity of the glandular layer; but a mild anatomical disease of the stomach may be present without causing a noteworthy change in the amount of pepsin. An excess of pepsin may be secreted and is often met with in chronic hypersthenic gastritis. The early morning contents of continuous secretion often digest more rapidly than the contents obtained after a test-meal given to the same individual. Whenever the filtrate of the early morning contents possesses greater digestive power than the filtrate of the test-breakfast contents, there is motor insufficiency. In simple continuous secretion, the early morning contents possess no greater digestive power than the contents after the test-breakfast.

In still another condition the pepsin tests are valuable, as when there is persistent and progressive diminution of the specific elements of secretion. This is a physical sign of chronic asthenic gastritis, of atrophy of the gastric glands or of carcinoma. It may be well to know, however, that the diminution of pepsin is not characteristic of any particular disease of the stomach and its quantity varies in very close relation with the quantity of total HCl. Consequently, its increase or decrease or its presence in normal quantity in the contents after the test-breakfast possesses about the same significance as like states of HCl secretion. It is also well to now that the loss of labferment secretion is of more grave significance than that of hydrochloric acid and pepsin secretion.

It is well next to consider the motor function of the stomach. The gastric muscle plays an exceedingly important part in the pathology of the stomach. Motor insufficiency is always indicative of more or less serious conditions. The chemical work of the stomach may be null without affecting nutrition if the integrity of the motor functions are maintained and the contents of the stomach are given over to the healthy intestines for assimilation and digestion. The intestinal juices are much more powerful and active than the gastric secretion.

Of the movements of the stomach there are two, that of churning and that of evacuation. These processes are undoubtedly due to the excitation of the ganglia contained in the stomach walls. During the first period of digestion, the duration of which is dependent upon the physiological action of the food, the tonic contraction predominates and the gastric wall applies itself closely to the gastric contents. These processes are necessary for the mixing of the various contents.

The second period is that of the worm-like movements, which are cardiac, pyloric or total, according to the location and extent of their visible expression. The normal motor function is dependent on a proper nerve supply, on a healthy muscle and on a physiological excitation. Consequently, motor function may be deranged through the central and sympathetic nerves by a badly nourished, weak or diseased gastric muscle and by improper contents in the stomach. It may also be hindered by adhesions, deformities displacements and obstructions to the evacuation of the stomach. Various methods have been devised for testing the motor function. One of these is by obtaining the contents of a given meal and determining the amount of the contents present at different periods during digestion, and comparing this with the amount normally present.

Another method is the use of salol. Salol is insoluble in the normal gastric juice and is decomposed in the intestine into carbolic and salicylic acids. The latter undergoes absorption and is eliminated as salicyluric acid in the urine. Normally, this may be detected in the urine in about sixty minutes, sometimes in thirty. The longer lapse of time before detection in the urine, the greater is motor insufficiency.

Another method is by the splashing sounds. Splashing throughout the period of digestion from day to day is a sign that the stomach muscles do not properly contract on its contents and perform its churning work. Another method is to direct the patient to eat a plate of soup, one roll and a beefsteak. Seven hours later the stomach should be normally empty. If the stomach is washed out at this time and any notable residue of the meal obtained, the stomach has failed to evacuate itself within the proper period. This failure may be due to insufficient secretion. The motor signs display a normal muscular activity and work or reveal some pathological variation. The pathological variations are excessive activity and insufficiency. Excessive activity is manifested by spasm-cardiospasm, gastrospasm, spasms of the pylorus, peristaltic unrest. Insufficiency is due either to a diminution of muscular power or to inability to overcome some mechanical interference.

In regard to the evacuation of the stomach, it may be too rapid or too slow. Too rapid occurs in pyloric organic diseases, particularly cancer which has possibly diverted the pylorus into a rigid, open canal. Delayed evacuation is most commonly the result of myasthenia. The other causes of delayed evacuation, when it is not a mere temporary condition or due to excessive secretion are the anatomical diseases already mentioned. The motor signs are all symptoms; not one of them is a disease.

One other change, absorption takes place through the stomach, but as not very much is known about it in the present state of our knowledge, it would not be profitable to discuss the subject, excepting to say that alcohol, the sugars and albumoses are absorbed in small quantities.

The chemical transformation of the food by the gastric juices and the saliva represent the amount of digestion by the stomach. The carbohydrates are not digested normally by the stomach but in some diseased conditions the saliva may act somewhat on them. The digestion of proteids can be determined by the tests given above.

